# *N*′-(4-Bromo­benzyl­idene)isonicotino­hydrazide

**DOI:** 10.1107/S1600536808036271

**Published:** 2008-11-13

**Authors:** Li-Min Li, Fang-Fang Jian

**Affiliations:** a Microscale Science Institute, Weifang University, Weifang 261061, People’s Republic of China

## Abstract

The title compound, C_13_H_10_BrN_3_O, was prepared by the reaction of isonicotinohydrazide and 4-bromo­benzaldehyde. The dihedral angle between the benzene and pyridine rings is 8.60 (12)°. The crystal packing is stabilized by inter­molecular C—H⋯O and N—H⋯O hydogen-bonding inter­actions.

## Related literature

For background on Schiff bases, see: Chiu *et al.* (1998 ▶). For comparative bond-length data, see: Cimerman *et al.* (1997 ▶). 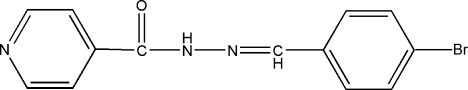


## Experimental

### 

#### Crystal data

C_13_H_10_BrN_3_O
*M* *_r_* = 304.15
Monoclinic, 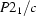

*a* = 18.715 (9) Å
*b* = 6.517 (3) Å
*c* = 10.126 (5) Å
β = 95.512 (9)°
*V* = 1229.3 (11) Å^3^
*Z* = 4
Mo *K*α radiation
μ = 3.33 mm^−1^
*T* = 273 (2) K
0.25 × 0.20 × 0.18 mm

#### Data collection

Bruker SMART CCD area-detector diffractometer
Absorption correction: none
7721 measured reflections
2992 independent reflections
1914 reflections with *I* > 2σ(*I*)
*R*_int_ = 0.034

#### Refinement

*R*[*F* ^2^ > 2σ(*F* ^2^)] = 0.036
*wR*(*F* ^2^) = 0.094
*S* = 1.01
2992 reflections
164 parameters
H-atom parameters constrained
Δρ_max_ = 0.39 e Å^−3^
Δρ_min_ = −0.55 e Å^−3^

### 

Data collection: *SMART* (Bruker, 1997 ▶); cell refinement: *SAINT* (Bruker, 1997 ▶); data reduction: *SAINT*; program(s) used to solve structure: *SHELXS97* (Sheldrick, 2008 ▶); program(s) used to refine structure: *SHELXL97* (Sheldrick, 2008 ▶); molecular graphics: *SHELXTL* (Sheldrick, 2008 ▶); software used to prepare material for publication: *SHELXTL*.

## Supplementary Material

Crystal structure: contains datablocks global, I. DOI: 10.1107/S1600536808036271/at2659sup1.cif

Structure factors: contains datablocks I. DOI: 10.1107/S1600536808036271/at2659Isup2.hkl

Additional supplementary materials:  crystallographic information; 3D view; checkCIF report

## Figures and Tables

**Table 1:** Hydrogen-bond geometry (Å, °)

*D*—H⋯*A*	*D*—H	H⋯*A*	*D*⋯*A*	*D*—H⋯*A*
N2—H2*A*⋯O1^i^	0.86	2.14	2.966 (3)	161
C12—H12*A*⋯O1^i^	0.93	2.60	3.377 (3)	142

Symmetry code: (i) 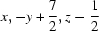.

**Fig. 1.**
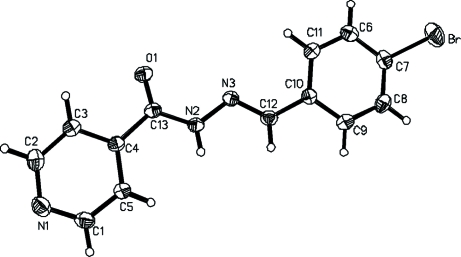
An ORTEP view of the title compound (I), showing 30% probability displacement ellipsoids and the atom-numbering scheme.

## supplementary crystallographic information

### Comment

Schiff bases have received considerable attention in the literature. They are attractive from several points of view, such as the possibility of analytical application (Cimerman *et al.*, 1997). As part of our search for new schiff base compounds we synthesized the title compound (I), and herein we report the crstal structure of (I).

In (I) (Fig. 1),

As seen in Fig. 1, the C12—N3 bond length of 1.276 (3)Å is comparable with C—N double bond [1.284 (2) Å] reported (Chiu *et al.*, 1998). In the title molecule, the benzene ring (C6–C10) is essentialy planar with a maximum deviation of 0.009 (2) Å for C6 and C9, while the pyridine ring is planar, with a maximum deviation of 0.012 (2) Å for C3. The dihedral angle between the benzene and pyridine rings is 8.60 (12)°.

The crystal packing is stabilized by intermolecular C—H···O and N—H···O hydogen-bonding interactions.

### Experimental

A mixture of the isonicotinohydrazide (0.1 mol), and 4-bromobenzaldehyde (0.1 mol) was stirred in refluxing ethanol (20 mL) for 4 h to afford the title compound (0.082 mol, yield 82%). Single crystals suitable for X-ray measurements were obtained by recrystallization from ethanol at room temperature.

### Refinement

H atoms were fixed geometrically and allowed to ride on their attached atoms, with N—H = 0.86 Å and C—H = 0.93 Å, and with *U*_iso_=1.2–*U*_eq_(C,N).

All H atoms were placed in idealized positions and refined with riding constraints, with N—H = 0.86 Å and C—H = 0.93 Å and with *U*_iso_(H) = 1.2*U*_eq_(C,N).

### Crystal data

**Table:**

C_13_H_10_BrN_3_O	*F*_000_ = 608
*M**_r_* = 304.15	*D*_x_ = 1.643 Mg m^−^^3^
Monoclinic, *P*2_1_/*c*	Mo *K*α radiation λ = 0.71073 Å
Hall symbol: -P 2ybc	Cell parameters from 2167 reflections
*a* = 18.715 (9) Å	θ = 3.3–24.3º
*b* = 6.517 (3) Å	µ = 3.33 mm^−^^1^
*c* = 10.126 (5) Å	*T* = 273 (2) K
β = 95.512 (9)º	Block, yellow
*V* = 1229.3 (11) Å^3^	0.25 × 0.20 × 0.18 mm
*Z* = 4	

### Data collection

**Table:**

Bruker SMART CCD area-detector diffractometer	1914 reflections with *I* > 2σ(*I*)
Radiation source: fine-focus sealed tube	*R*_int_ = 0.034
Monochromator: graphite	θ_max_ = 28.4º
*T* = 273(2) K	θ_min_ = 2.2º
φ and ω scans	*h* = −18→25
Absorption correction: none	*k* = −7→8
7721 measured reflections	*l* = −13→13
2992 independent reflections	

### Refinement

**Table:**

Refinement on *F*^2^	Hydrogen site location: inferred from neighbouring sites
Least-squares matrix: full	H-atom parameters constrained
*R*[*F*^2^ > 2σ(*F*^2^)] = 0.036	*w* = 1/[σ^2^(*F*_o_^2^) + (0.0429*P*)^2^ + 0.1134*P*] where *P* = (*F*_o_^2^ + 2*F*_c_^2^)/3
*wR*(*F*^2^) = 0.094	(Δ/σ)_max_ < 0.001
*S* = 1.01	Δρ_max_ = 0.39 e Å^−^^3^
2992 reflections	Δρ_min_ = −0.55 e Å^−^^3^
164 parameters	Extinction correction: SHELXL97 (Sheldrick, 2008), Fc^*^=kFc[1+0.001xFc^2^λ^3^/sin(2θ)]^-1/4^
Primary atom site location: structure-invariant direct methods	Extinction coefficient: 0.0072 (10)
Secondary atom site location: difference Fourier map	

### Special details

**Table:**

Geometry. All e.s.d.'s (except the e.s.d. in the dihedral angle between two l.s. planes) are estimated using the full covariance matrix. The cell e.s.d.'s are taken into account individually in the estimation of e.s.d.'s in distances, angles and torsion angles; correlations between e.s.d.'s in cell parameters are only used when they are defined by crystal symmetry. An approximate (isotropic) treatment of cell e.s.d.'s is used for estimating e.s.d.'s involving l.s. planes.
Refinement. Refinement of *F*^2^ against ALL reflections. The weighted *R*-factor *wR* and goodness of fit *S* are based on *F*^2^, conventional *R*-factors *R* are based on *F*, with *F* set to zero for negative *F*^2^. The threshold expression of *F*^2^ > σ(*F*^2^) is used only for calculating *R*-factors(gt) *etc*. and is not relevant to the choice of reflections for refinement. *R*-factors based on *F*^2^ are statistically about twice as large as those based on *F*, and *R*- factors based on ALL data will be even larger.

### Fractional atomic coordinates and isotropic or equivalent isotropic displacement parameters (Å^2^)

**Table:**

	*x*	*y*	*z*	*U*_iso_*/*U*_eq_	
Br	0.038461 (17)	0.64201 (4)	0.85386 (4)	0.08031 (18)	
N3	0.25010 (10)	1.5120 (3)	0.93706 (17)	0.0390 (4)	
N2	0.29511 (10)	1.6713 (3)	0.90925 (18)	0.0386 (4)	
H2A	0.3079	1.6847	0.8304	0.046*	
O1	0.30311 (10)	1.7983 (3)	1.11880 (16)	0.0529 (4)	
C7	0.10215 (13)	0.8680 (3)	0.8543 (3)	0.0457 (6)	
C10	0.19040 (12)	1.2072 (3)	0.8503 (2)	0.0369 (5)	
N1	0.46415 (11)	2.2729 (3)	0.8926 (2)	0.0545 (6)	
C4	0.36913 (11)	1.9665 (3)	0.9610 (2)	0.0349 (5)	
C13	0.31906 (12)	1.8058 (3)	1.0047 (2)	0.0362 (5)	
C9	0.19163 (13)	1.0539 (4)	0.7549 (2)	0.0446 (6)	
H9A	0.2229	1.0656	0.6893	0.054*	
C12	0.23656 (13)	1.3861 (3)	0.8414 (2)	0.0417 (5)	
H12A	0.2570	1.4094	0.7626	0.050*	
C3	0.37459 (13)	2.1531 (3)	1.0273 (2)	0.0448 (6)	
H3B	0.3471	2.1787	1.0972	0.054*	
C5	0.41305 (13)	1.9365 (4)	0.8605 (2)	0.0455 (6)	
H5A	0.4115	1.8140	0.8133	0.055*	
C11	0.14423 (13)	1.1841 (4)	0.9499 (2)	0.0438 (6)	
H11A	0.1429	1.2844	1.0150	0.053*	
C8	0.14730 (14)	0.8850 (3)	0.7560 (3)	0.0488 (6)	
H8A	0.1480	0.7843	0.6911	0.059*	
C6	0.10088 (13)	1.0151 (4)	0.9526 (2)	0.0473 (6)	
H6A	0.0708	0.9994	1.0199	0.057*	
C2	0.42134 (14)	2.3003 (4)	0.9882 (3)	0.0515 (6)	
H2B	0.4229	2.4263	1.0315	0.062*	
C1	0.45933 (14)	2.0919 (4)	0.8314 (3)	0.0552 (7)	
H1B	0.4891	2.0684	0.7646	0.066*	

### Atomic displacement parameters (Å^2^)

**Table:**

	*U*^11^	*U*^22^	*U*^33^	*U*^12^	*U*^13^	*U*^23^
Br	0.0729 (3)	0.04607 (19)	0.1216 (4)	−0.01855 (14)	0.0075 (2)	−0.00034 (16)
N3	0.0430 (11)	0.0399 (10)	0.0350 (10)	−0.0045 (8)	0.0083 (8)	0.0038 (9)
N2	0.0456 (11)	0.0415 (10)	0.0301 (10)	−0.0063 (8)	0.0103 (8)	0.0034 (8)
O1	0.0674 (12)	0.0593 (10)	0.0341 (9)	−0.0110 (9)	0.0160 (8)	−0.0036 (8)
C7	0.0409 (13)	0.0333 (12)	0.0618 (16)	0.0013 (9)	−0.0016 (12)	0.0032 (11)
C10	0.0383 (12)	0.0367 (11)	0.0352 (12)	0.0003 (9)	0.0013 (10)	0.0032 (10)
N1	0.0568 (14)	0.0567 (14)	0.0492 (13)	−0.0143 (11)	0.0019 (11)	0.0077 (11)
C4	0.0363 (12)	0.0387 (12)	0.0294 (11)	0.0036 (9)	0.0022 (9)	0.0023 (9)
C13	0.0380 (13)	0.0390 (11)	0.0321 (12)	0.0051 (9)	0.0055 (10)	0.0024 (10)
C9	0.0509 (14)	0.0462 (13)	0.0375 (13)	0.0051 (11)	0.0081 (11)	−0.0005 (11)
C12	0.0469 (14)	0.0440 (13)	0.0355 (12)	−0.0016 (10)	0.0102 (11)	0.0037 (11)
C3	0.0457 (14)	0.0457 (14)	0.0437 (13)	0.0035 (10)	0.0086 (11)	−0.0041 (11)
C5	0.0482 (14)	0.0493 (13)	0.0400 (13)	−0.0052 (11)	0.0097 (11)	−0.0050 (11)
C11	0.0472 (14)	0.0443 (13)	0.0403 (13)	−0.0005 (10)	0.0060 (11)	−0.0057 (10)
C8	0.0571 (16)	0.0396 (13)	0.0487 (15)	0.0076 (11)	0.0005 (13)	−0.0090 (11)
C6	0.0431 (14)	0.0510 (14)	0.0489 (14)	−0.0025 (11)	0.0103 (11)	0.0065 (12)
C2	0.0532 (16)	0.0402 (13)	0.0596 (16)	−0.0022 (11)	−0.0029 (14)	−0.0006 (12)
C1	0.0541 (16)	0.0706 (18)	0.0428 (14)	−0.0117 (13)	0.0139 (12)	0.0010 (13)

### Geometric parameters (Å, °)

**Table:**

Br—C7	1.894 (2)	C4—C13	1.500 (3)
N3—C12	1.276 (3)	C9—C8	1.379 (3)
N3—N2	1.383 (2)	C9—H9A	0.9300
N2—C13	1.349 (3)	C12—H12A	0.9300
N2—H2A	0.8600	C3—C2	1.382 (3)
O1—C13	1.222 (3)	C3—H3B	0.9300
C7—C8	1.371 (4)	C5—C1	1.382 (3)
C7—C6	1.384 (3)	C5—H5A	0.9300
C10—C9	1.391 (3)	C11—C6	1.370 (3)
C10—C11	1.398 (3)	C11—H11A	0.9300
C10—C12	1.459 (3)	C8—H8A	0.9300
N1—C2	1.326 (3)	C6—H6A	0.9300
N1—C1	1.331 (3)	C2—H2B	0.9300
C4—C5	1.382 (3)	C1—H1B	0.9300
C4—C3	1.388 (3)		
			
C12—N3—N2	114.10 (18)	C10—C12—H12A	118.6
C13—N2—N3	120.59 (18)	C4—C3—C2	119.3 (2)
C13—N2—H2A	119.7	C4—C3—H3B	120.4
N3—N2—H2A	119.7	C2—C3—H3B	120.4
C8—C7—C6	121.4 (2)	C4—C5—C1	118.9 (2)
C8—C7—Br	119.52 (19)	C4—C5—H5A	120.6
C6—C7—Br	119.09 (19)	C1—C5—H5A	120.6
C9—C10—C11	118.4 (2)	C6—C11—C10	120.6 (2)
C9—C10—C12	118.8 (2)	C6—C11—H11A	119.7
C11—C10—C12	122.8 (2)	C10—C11—H11A	119.7
C2—N1—C1	116.1 (2)	C7—C8—C9	118.9 (2)
C5—C4—C3	117.3 (2)	C7—C8—H8A	120.5
C5—C4—C13	123.5 (2)	C9—C8—H8A	120.5
C3—C4—C13	119.2 (2)	C11—C6—C7	119.5 (2)
O1—C13—N2	123.8 (2)	C11—C6—H6A	120.3
O1—C13—C4	121.6 (2)	C7—C6—H6A	120.3
N2—C13—C4	114.59 (18)	N1—C2—C3	124.0 (2)
C8—C9—C10	121.2 (2)	N1—C2—H2B	118.0
C8—C9—H9A	119.4	C3—C2—H2B	118.0
C10—C9—H9A	119.4	N1—C1—C5	124.4 (2)
N3—C12—C10	122.9 (2)	N1—C1—H1B	117.8
N3—C12—H12A	118.6	C5—C1—H1B	117.8

### Hydrogen-bond geometry (Å, °)

**Table:**

*D*—H···*A*	*D*—H	H···*A*	*D*···*A*	*D*—H···*A*
N2—H2A···O1^i^	0.86	2.14	2.966 (3)	161
C12—H12A···O1^i^	0.93	2.60	3.377 (3)	142

Symmetry codes: (i) *x*, −*y*+7/2, *z*−1/2.
